# High-Level Disinfection in Ambulatory Care: Overcoming the Barriers of a Decentralized System through Auditing and Education

**DOI:** 10.1017/ash.2021.21

**Published:** 2021-07-29

**Authors:** Sonja Rivera Saenz

## Abstract

**Background:** High-level disinfection (HLD) of semicritical instruments in a multispecialty ambulatory care network has the potential for increased risk due to the decentralized instrument reprocessing and lack of a sterile processing department. Attention to HLD practices is an important part of device-borne outbreak prevention. **Method:** An HLD database was developed to identify specific departments and locations where HLD occurred across a 30-medical practice ambulatory care network in eastern Massachusetts, which included otolaryngology, urology, endoscopy, and obstetrics/gynecology departments. Based on qualitative feedback from managers and reprocessing staff, this database centralized information that included the supply inventory including manufacturer and model information, HLD methodology, standard work, and listing of competency evaluations. The infection control team then led audits to directly observe compliance with instrument reprocessing and a monthly-driven HLD calendar was developed to enforce annual competencies. **Result:** The results of the audits demonstrated variability across departments with gaps in precleaning, transportation of used instruments, the dilution of enzymatic cleaner, and maintenance of quality control logs. Given the uniqueness of shape and size of various ambulatory locations, proper storage and separation between clean and dirty spaces were common pitfalls. Auditing also revealed different levels of staff understanding of standard work and variable inventory management. Centralized education sessions held jointly by the infection control team and various manufacturers for the reprocessing staff helped to create and reinforce best practices. **Conclusion:** Decentralized HLD that occurs across multiple ambulatory care sites led to gaps in instrument reprocessing and unique challenges due to variable geography of sites, physical space constraints, and an independent approach to procuring medical supplies. Through the auditing and feedback of all areas that perform HLD, an effective and sustainable strategy was created to ensure practice improvement. Streamlining standard work, seeking direct input from frontline staff, and collective educational events were critical to our success in the ambulatory setting.

**Funding:** No

**Disclosures:** None

